# Establishment and application of Agrobacterium-delivered CRISPR/Cas9 system for wild tobacco (*Nicotiana alata*) genome editing

**DOI:** 10.3389/fpls.2024.1329697

**Published:** 2024-03-04

**Authors:** Cheng Yuan, Jianmin Zeng, Yong Liu, Haiqin Yu, Zhijun Tong, Jianduo Zhang, Qian Gao, Zhong Wang, Xueyi Sui, Bingguang Xiao, Changjun Huang

**Affiliations:** ^1^ Yunnan Academy of Tobacco Agricultural Sciences, Key Laboratory of Tobacco Biotechnological Breeding, National Tobacco Genetic Engineering Research Center, Kunming, China; ^2^ Technology Center, China Tobacco Yunnan Industrial Co. LTD, Kunming, China; ^3^ China Tobacco Gene Research Center, Zhengzhou Tobacco Research Institute of CNTC, Zhengzhou, China

**Keywords:** genome editing, CRISPR/Cas9, wild tobacco, *Nicotiana alata*, self-incompatibility

## Abstract

Clustered regularly interspaced short palindromic repeats (CRISPR)-associated protein 9 (CRISPR-Cas9) system has been widely applied in cultivated crops, but limited in their wild relatives. *Nicotiana alata* is a typical wild species of genus *Nicotiana* that is globally distributed as a horticultural plant and well-studied as a self-incompatibility model. It also has valuable genes for disease resistance and ornamental traits. However, it lacks an efficient genetic transformation and genome editing system, which hampers its gene function and breeding research. In this study, we developed an optimized hypocotyl-mediated transformation method for CRISPR-Cas9 delivery. The genetic transformation efficiency was significantly improved from approximately 1% to over 80%. We also applied the CRISPR-Cas9 system to target the *phytoene desaturase* (*NalaPDS*) gene in *N. alata* and obtained edited plants with PDS mutations with over 50% editing efficiency. To generate self-compatible *N. alata* lines, a polycistronic tRNA-gRNA (PTG) strategy was used to target exonic regions of allelic *S-RNase* genes and generate targeted knockouts simultaneously. We demonstrated that our system is feasible, stable, and high-efficiency for *N. alata* genome editing. Our study provides a powerful tool for basic research and genetic improvement of *N. alata* and an example for other wild tobacco species.

## 1 Introduction

Crop wild relatives (CWRs) harbor untapped genetic diversity and resilience, serving as a genetic reservoir to improve resistance, yield, quality and adaptability of cultivated crops. However, during the process of domestication, with the main purpose of increasing crop yield and biomass, a treasure trove of genes was lost in cultivated crops (Sang et al., 2011; Tirnaz et al., 2022). As the demands of a burgeoning global population escalate alongside the multifaceted challenges posed by environmental stresses, scientists have turned to explore the key functional genes that control beneficial traits in CWRs and are lost in cultivated crops, and applied them to molecular breeding (Bohra et al., 2022). Consequently, the modern agricultural paradigm is undergoing a transformation from concentrating on staple crops to interplaying between cultivated crops and their wild progenitors. In recent decades, several ‘game-changing’ genes that can offer beneficial traits, such as resistance to pests and diseases, abiotic stress tolerance, quality improvements and yield increases, have been reintroduced into cultivated varieties to enhance their resilience and yield (Engels and Thormann, 2020). Growing evidence has also demonstrated the value of CWRs in agricultural breeding programs, with the annual contribution of these traits to agriculture estimated at USD 115 billion globally, but they still remain a relatively under-utilized and under-estimated resource (Dempewolf et al., 2017). Many valuable traits have not yet been explored and most of the important functional genes in CWRs have not yet been studied.

With the rapid development of sequencing technology and the sharp decrease of sequencing cost, the genomic information of important CWRs has been uncovered in the last decade (Weigel and Nordborg, 2015; Purugganan and Jackson, 2021). Integrated with large-scale transcriptome, metabolome and/or proteome information, candidate genes that determine important traits are now readily predicted. To maximize these potential valuable assets, candidate gene validation and function identification by using genetic engineering approaches, such as gene overexpression, gene silencing and gene knockout mediated by genetic transformation methods, could facilitate the deployment of wild alleles into new cultivars. However, in contrast to the well-developed systems in the cultivars, efficient genetic transformation systems have not yet been established in amounts of the CWRs (Chen et al., 2022).

Clustered regularly interspaced short palindromic repeats (CRISPR)-associated protein (CRISPR-Cas) based genome editing technology is a recently developed powerful tool and has been widely utilized in plant molecular biology (Manghwar et al., 2019; Zhu et al., 2020). Compared to the conventional strategies used for functional characterization of plant genes and genetic improvement of agricultural crops, which are complicated and laborious-, time-consuming, CRISPR-Cas, especially CRISPR-Cas9 from *Streptococcus pyogenes*, has been proved in plant biology and provides more effective and time-saving methods with precise genetic manipulation of target genes (Hsu et al., 2014; Liu et al., 2016). However, while CRISPR-Cas9 has enabled various genome editing applications, such as indels production, precise nucleotide substitution, gene-expression regulation, multiplexed and high-throughput gene editing in a variety of model plants and crop species, the recalcitrance of efficient genetic transformation severely hinders the application of CRISPR-Cas9 in the CWRs (Rahman et al., 2023). Consequently, due to the lack of efficient CRISPR-Cas9 editing system, gene function verification of most CWRs *in situ* still cannot be carried out.

*Nicotiana alata* is a perennial species native to Brazil, Paraguay, and northeastern Argentina, belonging to the genus *Nicotiana* section Alatea. It has been introduced to other regions as an ornamental plant for its attractive and fragrant flowers that vary in color (Zheng et al., 2021; Zhang et al., 2023). As an important horticultural plant, the genes involved in the secondary metabolism pathway that regulate the fragrance and color of the flowers need to be further addressed (Raguso et al., 2003, 2006; Fahnrich et al., 2011; Guo et al., 2020; Zheng et al., 2021). In its native wild habitats, *N. alata* encounters a range of biotic and abiotic stresses. To survive these challenges, it has evolved a suite of adaptive systems that confer protection against specific threats. Several resistance loci have been identified in *N. alata*, including but not limited to, resistance to *Tomato spotted wilt virus* (*RTSW*), *Tobacco mosaic virus* (*N’ala*) and black root rot (Yuan et al., 2015; Trojak-Goluch et al., 2016; Huang et al., 2018; Berbeć and Doroszewska, 2020; Czubacka, 2022; Li et al., 2023). More important, *N. alata* is a model species used to study gametophytic self-incompatibility (SI), controlled by the *S*-locus (Bredemeijer and Blaas, 1981). The first cloning and sequencing of a cDNA encoding an S-RNase protein that co-segregates with an *S* allele was obtained in *N. alata* (Anderson et al., 1986; Mcclure et al., 1989). The *S*-locus gene of *N. alata* has subsequently been extensively studied by molecular biologists and geneticists, as it provides a model system to understand the molecular mechanisms of SI, as well as its evolution, diversity, and plasticity (Cruz-Garcia et al., 2003, 2005; Roldan et al., 2015; Fujii et al., 2016). In addition, the interaction between *N. alata* and its pollinator, the hawkmoth *Manduca sexta*, is also a fascinating example of coevolution and mutualism (Kessler et al., 2015; Haverkamp et al., 2016). In summary, the diverse roles and significance of *N. alata* in research, from reproductive biology to environmental interactions, underline it is not only an ornamental plant but also a model organism.

Despite its pivotal role in scientific inquiries, researchers working with *N. alata* faced a significant technological hurdle. The most significant challenge posed by *N. alata*, particularly its recalcitrance to conventional genetic transformation techniques like the leaf disk method (Ebert and Clarke, 1990; Taheri-Dehkordi et al., 2017), has been a major obstacle for researchers. The lack of highly efficient genetic transformation has led to the revolutionary CRISPR/Cas9 genome-editing system, which has transformed genetic research in myriad organisms, but has not been established in *N. alata*. In this study, we optimized the approach centered on hypocotyl transformation. This innovative shift not only circumvented the longstanding challenges but also set the stage for new genome-editing endeavors in *N. alata*. By using the *phytoene desaturase* (*PDS*) gene as a proof of concept, we validated the efficacy of our CRISPR-Cas9 system in *N. alata*. For further breeding and basic research in future, we aimed to disrupt the *S-RNase* gene and thereby obtain self-compatible *N. alata*. Therefore, we not only established an efficient genome editing system but also obtained an important germplasm for future study.

## 2 Materials and methods

### 2.1 Cloning of the first exon of the *N. alata PDS* (*NalaPDS*) coding sequence

The annotated *PDS* sequences of *N. tabacum* (XM_016642616), *N. benthamiana* (EU165355), and *N. attenuata* (JX185751) were retrieved from the National Centre for Biotechnology Information (NCBI) database and used as queries for a Basic Local Alignment Search Tool-Nucleotide (BLASTn) search against the draft genome sequences of *N. tabacum*, *N. benthamiana*, and *N. attenuata*, respectively (available at https://solgenomics.net). The obtained genomic DNA and coding sequences (CDs) were aligned (**Supplementary Figure 1**) using the MEGA software (Tamura et al., 2021). Primer pairs, NalaPDS1stExon-F/-R (**Supplementary Table 1**), were designed based on the conserved regions in the exonic region to isolate the partial *PDS* coding sequence from *N. alata*. Total RNA was extracted from *N. alata* leaf tissue using the RNeasy Plant Mini Kit (Qiagen, CA, USA) and the first strand of cDNA was synthesized using The PrimeScript 1st strand cDNA Synthesis Kit (Takara, Dalian, China) according to the manufacturer’s instructions. Polymerase chain reaction (PCR) amplification was carried out using 100 ng of cDNA as a template and the Q5 High-Fidelity DNA Polymerase, following the recommended cycling conditions provided by the manufacturer (NEB, MA, USA). The PCR amplification consisted of an initial denaturation step at 94°C for 2 minutes, followed by 30 cycles of denaturation at 94°C for 30 seconds, annealing at 60°C for 30 seconds, and extension at 72°C for 1 minute. The amplified product was recovered from a low-melting point agarose gel and subsequently sequenced.

### 2.2 *S-RNase* allele identification in *N. alata*

To identify the *S-RNase* allele in *N. alata*, degenerate primers C2F/C4R (**Supplementary Table 1**) were used to amplify the *S-RNase* candidates. Genomic DNA was extracted from *N. alata* leaf tissue using the Plant Genomic DNA Miniprep Kit protocol (Tiangen, Tianjin, China). Genomic PCR was performed using the Q5 High-Fidelity DNA Polymerase, as described above. The amplified PCR product was cloned into the pCE2-TA-Blunt-Zero vector (Vazyme, Nanjing, China) following the manufacturer’s instructions. 10 Clones of each plant were sequenced using Sanger sequencing platform (Generay, Shanghai, China), and the obtained sequences were subjected to BLAST search against the non-redundant NCBI database to determine the candidate *S-RNase* allele.

To confirm the *S2/Sc10* bi-allele distribution result in the family, specific primer pairs S2F/R and Sc10F/R (**Supplementary Table 1**) were used to examine the same population. The PCR products displaying expected size unique bands were subjected to electrophoresis using the ZAG DNA Analyzer (Agilent, Santa Clara, USA).

### 2.3 Guide RNA design and plasmid construction

Cas9-PF vector was digested using the *Bsa*I enzyme (NEB) for vector linearization. The online CRISPR software (http://crispor.tefor.net/) was utilized to identify sgRNA sequences in the gene’s first exon (Concordet and Haeussler, 2018). For *NalaPDS*, the oligonucleotides of sgRNA (**Supplementary Table 1**) were annealed and ligated into *Bsa*I-digested Cas9-PF plasmids to generate the Cas9-PF-NalaPDS structure. For *S-RNase*, a polycistronic tRNA-gRNA (PTG) cassette (**Supplementary Table 1**) containing S2-RNase and Sc10-RNase gRNAs was synthesized (Generay, Shanghai, China). The synthesized PTG polynucleotides were inserted into *Bsa*I-digested Cas9-PF plasmids through homologous recombination to generate the Cas9-PF-SRNase structure.

### 2.4 Sterilization seeds preparation of *N. alata* plants

The *N. alata* plants (accession PI42334), used in this study, were provided by Prof. Hanhui Kuang (Huazhong Agriculture University) (Yuan et al., 2015). The seeds of the *N. alata* line were harvested after crossing from different plants. Sterilization of the seeds was carried out by placing them in a 1.5-mL microfuge tube with 70% ethanol for 1 minute, followed by treatment with a bleach solution containing 10% sodium hypochlorite for 10 minutes. After five rinses with sterilized deionized water, the sterilized seeds were sown on agar plates containing plant growth media (**Table 1**).

**Table 1:** Optimized steps, medium used and culture conditions in *Agrobacterium*-mediated transformation of *Nicotiana alata*.

Type of Medium	Medium content	Antibiotics applicaiton	Culture conditions	Culture time
Washing medium	MS basal medium (4.41 g/L), 6-Benzyladenine (6-BA) 1 mg/L, IAA 0.02 mg/l, 30 g/L sucrose, adjust the pH to 5.8 with 1 M KOH.	None	None	None
Co-cultivation	MS basal medium (4.41 g/L), 6-BA 1 mg/L, IAA 0.02 mg/l, 30 g/L sucrose, and 5 g/L Bacto agar, adjust the pH to 5.8 with 1 M KOH.	None	Dark, 25°C	3 days
Callus induction	MS basal medium (4.41 g/L), 6-BA 1 mg/L, IAA 0.02 mg/l, 30 g/L sucrose, and 5 g/L Bacto agar, adjust the pH to 5.8 with 1 M KOH.	Timentin (125 mg/L) for *Agrobacterium* inhibition and hygromycin (35 mg/L) for selectable antibiotic	Long-day condition (16-h light/8-h dark, 25 °C)	3-5 weeks
Shoot regeneration	MS basal medium (4.41 g/L), 6-BA 1 mg/L, 30 g/L sucrose, and 5 g/L Bacto agar, adjust the pH to 5.8 with 1 M KOH.	Timentin (125 mg/L) for *Agrobacterium* inhibition and hygromycin (35 mg/L) for selectable antibiotic	Long-day condition (16-h light/8-h dark, 25 °C)	3 weeks
Rooting	1/4 X MS basal medium (4.41 g/L), 0.8 mg/L 4-(3-Indolyl) butyric acid, 30 g/L sucrose, and 6 g/L Bacto agar, adjust the pH to 5.8 with 1 M KOH	Timentin (125 mg/L) for Agrobacterium inhibition	Long-day condition (16-h light/8-h dark, 25 °C)	3-5 weeks

### 2.5 *Agrobacterium* culture preparation

Target plasmids were transformed into *A. tumefaciens* using electroporation. For each transformation, 100 μl of competent cells were mixed with 50 ng of plasmid DNA and suspended in an electroporation cuvette with an electrode distance of 1 mm (Bio-Rad, USA). Electroporation was carried out using MicroPulser Electroporator (Bio-Rad, USA) with 2.5 kV, 25 μF capacitance, and 400 Ohm resistance. To screen for transformants of the target plasmid, colony PCR was performed using specific primers. Each colony was suspended in 20 μl of 1 X Taq DNA polymerase Master Mix (Vazyme, Nanjing, China) and subjected to PCR amplification. Positive single colonies were transferred to 50 mL centrifuge tubes containing 5 mL of liquid LB medium supplemented with 50 mg/L rifampicin and 100 mg/L kanamycin. The bacterial cultures were incubated on a shaker overnight at 220 rpm and 28°C until the OD at 600 nm (OD600) reached 0.8. The *Agrobacterium* cultures were then centrifuged at 2500 × g for 5 minutes, washed two times with MS medium containing 30 g/L sucrose. The bacterial pellet was resuspended in 4 mL of MS medium containing 30 g/L sucrose and supplemented with 100 µM acetosyringone to achieve an OD600 of 0.3 - 0.4.

### 2.6 *Agrobacterium* strains screening

To screen for the most efficient *Agrobacterium* strain, we performed the transformation using four *A. tumefaciens* strains: GV3101, C58C1, EHA105 and LBA4404. All strains harbored the same Cas9-PF-SRNase plasmid as described above. This plasmid contained the *hygromycin B phosphotransferase* gene as a selectable marker for plant selection (conferring hygromycin resistance) under the control of the 35S promoter and CaMV poly(A) signal terminator. We evaluated the transformation efficiency by measuring the callus proliferation and shoot regeneration rates.

### 2.7 Explants preparing and *Agrobacterium* infection

The sterilized seeds were germinated for 3 days in the dark (25 °C) and then transferred to long-day condition (16-h light/8-h dark, 25 °C) for another 7 days. The seedlings (15-20 mm in length) were excised below the apical meristem and above the roots. The hypocotyl part was subsequently cut into 4-5 segments (about 3–5 mm in length) and the segments were placed on cocultivation medium (**Table 1**). The hypocotyl preparing and *Agrobacterium* infection could be achieved simultaneously by dipping the scalpel into *Agrobacterium* suspension before each cut.

### 2.8 Callus induction and shoots regeneration

After 3 days of co-cultivation with *Agrobacterium*, explants were taken out and transferred to callus induction medium (**Table 1**) containing the antibiotic (cefotaxime or timentin) to inhibit growth of *Agrobacterium* and subsequently sub-cultured at second week. After 2-3 weeks, when explants had developed swelling callus with shoot primordia, the calli were transferred onto regeneration medium (**Table 1**), which contained both *Agrobacterium* and plant antibiotics. The calli with shoots were subsequently sub-cultured every 3 weeks until plantlets were formed. These plantlets were separated into single plants and cultured on rooting medium without selectable antibiotics (**Table 1**). At this stage, clean cut of callus from the base of the plantlet was essential. Plantlets were sub-cultured on rooting media every 3 weeks until roots developed. When the roots are 2-3 cm long, plants were carefully removed from the gel and transplanted into soil. These transgenic plants were subsequently maintained in growth chambers at long-day condition (16-h light/8-h dark, 25 °C).

### 2.9 Effect of antibacterial antibiotics on *Agrobacterium* growth

To identify the most suitable antibiotic and its concentrations for the *Agrobacterium* growth inhibition, different ranges of cefotaxime and timentin concentrations were evaluated according to the ones described in the literature for other *nicotiana* species. *Agrobacterium* infected hypocotyl explants were transferred into co-cultivation and callus induction plates containing MS medium with cefotaxime alone at concentrations of 50, 100, 200 and 300 mg/L and timentin alone at concentrations of 50, 125, 200 and 300 mg/L. Data were statement as the callus proliferation and shoot regeneration rate.

### 2.10 Detection of genome editing events

The genomic DNA of the stable transgenic *N. alata* plants from hygromycin selection and wild-type plants were extracted from leaf tissue using the Plant Genomic DNA Miniprep Kit protocol (Tiangen) to assess targeted mutagenesis using PCR amplification and Sanger sequencing. The genomic region spanning the CRISPR target sequences were amplified by PCR (primer sequences in **Supplementary Table 1**) using Q5 High-Fidelity DNA Polymerase (NEB). The PCR products were sequenced and then subjected to SnapGene software assay (www.snapgene.com) according to the manufacturer’s instructions. PCR fragments of putative editing events were then cloned into pCE2-TA-Blunt-Zero vector (Vazyme) and 10 clones each plant were sequenced to further measure the frequencies of CRISPR induced mutations. The mutation rate was calculated based on the ratio of mutated plants versus total transgenic plants.

## 3 Results

### 3.1 Optimization of *Agrobacterium*-mediated transformation and regeneration

Two methods of Agrobacterium-mediated transformation in *N. alata* have been previously described (Ebert and Clarke, 1990; Schroeder and Stimart, 1996). Leaf discs and seedling hypocotyls were utilized as explants for plantlet regeneration, respectively. Initially, we strictly followed the methodology described by Schroeder and Stimart (1996) for leaf discs-mediated transformation, as it is a conventional method applied in multiple *Nicotiana* species. However, our attempts were unsuccessful. We made efforts to adjust various parameters, including different *Agrobacterium* strains, selectable antibiotic concentrations, and callus and shoot induction media compositions, in order to achieve improved outcomes. Unfortunately, none of the tested parameters resulted in the formation of well-developed callus and regeneration shoots in *N. alata*. Instead, friable and disorganized tumors were observed (data not shown). Therefore, we speculated that the use of leaf explants may not be suitable for generating transgenic lines of our owned *N. alata* (PI42334) line.

**Figure 1:**
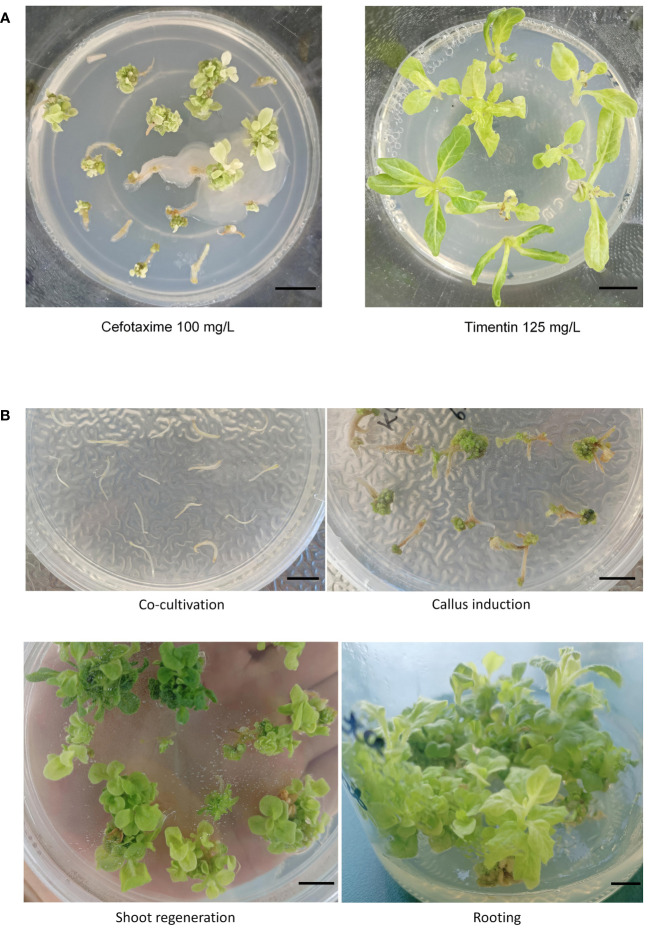
*Agrobacterium*-mediated transformation of *Nicotiana alata* using hypocotyl explants. **(A)** Inhibition of *Agrobacterium* growth by 100 mg/L cefotaxime and 125 mg/L timentin. **(B)** Regeneration frequency of *N. alata* based on optimized transformation method. Representative transformation steps are shown: hypocotyl segments were co-cultured with Cas9-PF-SRNase, callus formation and shoot regeneration on selection medium with hygromycin (35 mg/L) and timentin (125 mg/L), *in vitro* culture and transgenic seedling on root induction medium. Scale bars = 5 mm.

Although the regeneration of transformed *N. alata* using hypocotyl explants was established, the low transformation frequency (<1%) was identified as a bottleneck (Ebert and Clarke, 1990). To enhance the transformation frequency, we aimed to optimize the *Agrobacterium* strains, co-culture reagents. Four *Agrobacterium* tumefaciens strains, namely GV3101, C58C1, EHA105, and LBA4404, were tested to evaluate the effects of strain types on the transformation efficiency in *N. alata*. Hypocotyls from 10-day-old seedlings were cut into 3-5 mm segments as explants. The explants were then transferred to callus-induction medium (**Figures 1A, B**) and cultured in darkness for 2 days to increase infection efficiency before being transferred to a long-day condition (16-hour light/8-hour darkness, 26°C). After 2 weeks on the callus induction plates and one month on the regeneration induction plates, containing 100 mg/L cefotaxime and 35 mg/L hygromycin, the hypocotyl segments gave rise to calli and shoots. The results demonstrated successful transformation of *N. alata* using GV3101, EHA105, and LBA4404 (**Table 2**). *A. tumefaciens* EHA105 and GV3101 exhibited higher transformation efficiency, with 12.5% and 8.33% regeneration shoot, respectively. Consistent with previous studies by Ebert and Clarke (1990), LBA4404 showed successful transformation but low efficiency (2.08%) among the four strains, whereas C58C1 failed to transform *N. alata*.

**Table 2:** Transformation efficiency of callus and regenerated shoots by different *Agrobacterium* strain.

Agrobacterium strain	No. of explants	No. of resistant calli induction	No. of resistant shoots induction
GV3101	48	20	4
C58C1	48	0	0
EHA105	48	23	6
LBA4404	48	12	1

During the screening of *A. tumefaciens* strains, we found that the primary cause of low transformation efficiency was *Agrobacterium* contamination (**Figure 1A**). Therefore, we next investigated the impact of antibiotics on *Agrobacterium* during the transformation process. Cefotaxime, commonly used in tissue culture of *N. tabacum* and *N. benthamiana*, is known to effectively eliminate any remaining *Agrobacterium* after co-cultivation. However, our study on *N. alata* transformation revealed a conflicting relationship between calli/shoots induction and the anti-agrobacterial effect of cefotaxime (**Table 3**). Upon screening, bacterial growth was observed at concentrations below 200 mg/L of cefotaxime (**Table 3**). Conversely, concentrations higher than 200 mg/L exhibited effective antibacterial properties against surface-growing bacteria, but severely impaired calli induction and shoots regeneration (**Table 3**). Recent studies have demonstrated the efficacy of timentin in suppressing *A. tumefaciens*, comparable to carbenicillin and cefotaxime, with minimal impact on shoot regeneration (Cheng et al., 1998; Krügel et al., 2002). Hence, we explored the effects of timentin on calli induction, shoot regeneration of *N. alata* hypocotyls, and its inhibitory ability on *Agrobacterium* growth. As presented in **Table 3**, timentin alone exhibited more efficient bacteria elimination than cefotaxime. After two weeks in the presence of these antibiotics, calli induction on timentin plates with concentrations higher than 125 mg/L proliferated normally without observable negative effects. Our findings indicate that timentin, with its broad spectrum of concentrations for suppressing *A. tumefaciens* in *Agrobacterium*-mediated genetic transformation, enabled successful regeneration, whereas cefotaxime significantly hindered regeneration.

**Table 3:** Antibiotic effect screening on *Agrobacterium* EHA105.

Antibiotics	Concentrations (mg/L)	No. of explants	No of calli induction	No of calli without *Agrobacterium* growth	No of shoots induction	No. of resistant shoots without Agrobacterium growth
Cefotaxime	50	48	26	0	0	0
	100	48	32	11	8	5
	200	48	8	8	3	1
	300	48	0	0	0	0
Timentin	50	48	34	26	18	18
	125	48	43	43	40	40
	200	48	38	38	38	38
	300	50	24	24	16	16

After investigating different *Agrobacterium* strains and antibacterial antibiotics, we determined that genetic transformation using the EHA105 strain and timentin at a concentration of 125 mg/L resulted in higher values for *N. alata* calli induction and shoot regeneration. Ultimately, we summarized the optimized *Agrobacterium*-mediated transformation of *N. alata* with media composition used, culture conditions, and average duration in different steps in **Table 1**.

### 3.2 Cas9−induced mutagenesis of *NalaPDS* in *N. alata*

**Figure 2:**
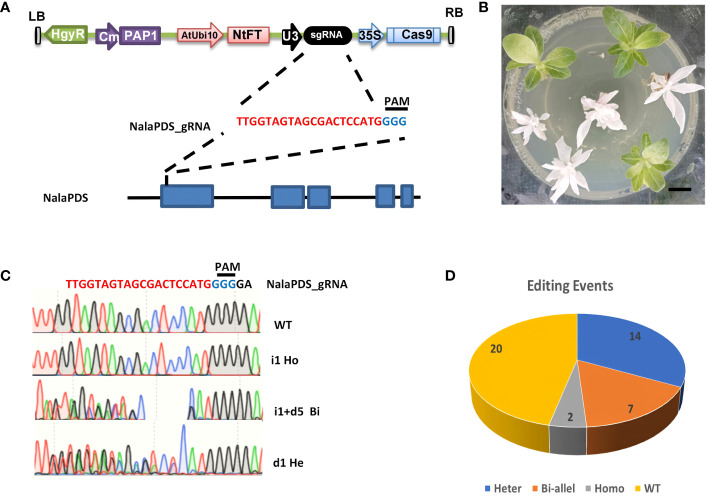
*Agrobacterium*-mediated CRISPR/Cas9 genome editing of *NalaPDS* in *N. alata*. **(A)** T-DNA constructs for Cas9-PF-NalaPDS. Cas9-PF-NalaPDS consists of a PF Cassette, which contains anthocyanin synthesis regulation gene *PAP1* and early flower *NtFT5* gene, as a visible marker, a *Cas9* gene driven by 35S promoter, an sgRNA targeting the *NalaPDS* gene under the control of a U3 promoter, and a hygromycin resistance gene. The first exon of the *N. alata* PDS gene was selected as the target site for the gRNA. **(B)** Phenotypes of the *pds* mutants. Seedlings displayed albino phenotype are homozygous or bi-allele gene editing of *NalaPDS*. Scale bar=1 cm. **(C)** PCR product sequencing results of PDS mutant plants in **(B)** are aligned to the reference genome sequence. Different chromatograms of target position indicate different types of editing events, e.g., homozygous (Ho), bi-allele (Bi) and heterozygous (He) type editing. WT, wild-type; inserts and deletions are indicated as ‘i’ and ‘d’ respectively. **(D)** Total precise editing events categorized by different editing types, including homozygous (Homo), bi-allele (Bi-allel) and heterozygous (Heter) and wild-type (WT).

The *PDS* gene is responsible for encoding one of the crucial enzymes involved in the carotenoid biosynthesis pathway. Mutant plants with an albino phenotype, resulting from disruptions in this gene, have been widely utilized as a model gene for virus-induced gene silencing and CRISPR/Cas9-mediated gene editing (Qin et al., 2007; Huang et al., 2009; Ma et al., 2020). Due to the absence of sequence information for the *PDS* gene in *N. alata* (referred to *NalaPDS*), conserved primers were designed based on the homologous nucleotide sequences of the *PDS* gene in *N. tabacum*, *N. benthamiana*, and *N. attenuate* (Edwards et al., 2017; Xu et al., 2017; Ranawaka et al., 2023). These primers were employed to amplify the *NalaPDS* partial CDs region from total RNA. Subsequently, a guide RNA (gRNA) targeting the first exon of *NalaPDS* was selected and integrated into the Cas9-PF system (Liu et al., 2019). The resulting construct (**Figure 2A**) was then introduced into *N. alata* plants using the optimized *Agrobacterium*-mediated genetic transformation protocol mentioned earlier.

Total 43 transformed seedlings were recovered with hygromycin selecting from 50 explants. Among them, 9 plants exhibited albino leaf phenotype which was recognized as completely disrupted *NalaPDS*, loss-of-function mutants (**Figure 2B**). Interestingly, the visual selection marker system mediated by the PAP gene in this CRISPR system (Liu et al., 2019) was ineffective in *N. alata*, none of the transformed seedlings exhibited purple phenotype in this study (**Figure 2B**). Specific primers were designed to amplify the target regions and sequenced to detect the mutant plants with *NalaPDS*. Mutations in *NalaPDS* were identified in 23 of 43 independent transgenic plants with the percentage of 53.5%. To further dissect the type of mutation, we also cloned and sequenced (10 random selected clones each plant) from the amplification of genomic DNA extracted from mutants (**Figure 2C**). We found that two and seven of nine displayed albino leaves are homozygous editing and bi-allele gene editing respectively and remaining 14 mutations in *NalaPDS* which show green leaf as wild type (WT) are heterozygous editing with indel on one allele (**Figure 2D**). Notably, the efficient mutations were generated in the *NalaPDS* locus of transgenic plants with indel rates more than 50% at T0 generation. These results showed that the CRISPR/Cas9 system could be used to modify genome and have high efficiency for targeted mutagenesis in *N. alata*.

### 3.3 Targeted mutagenesis of *S-RNase* results in self-compatibility

**Figure 3:**
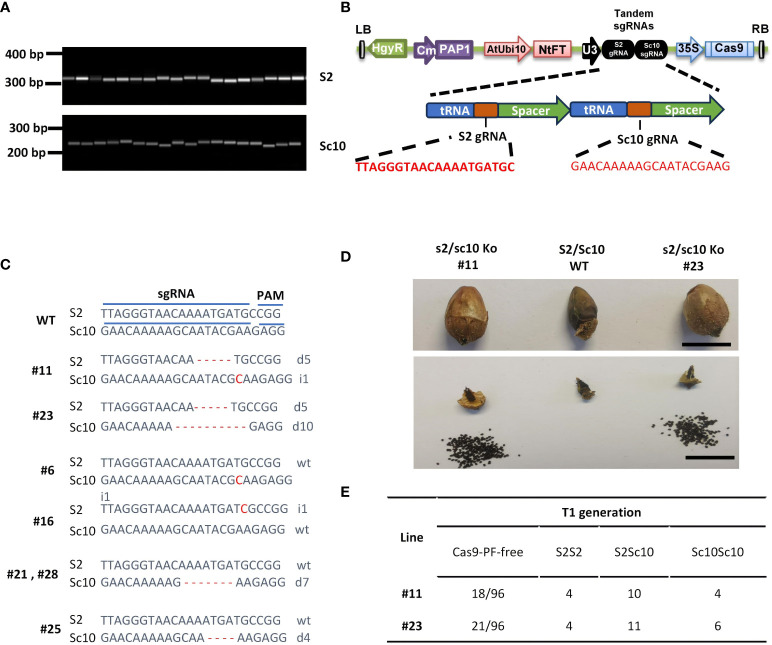
CRISPR/Cas9-mediated *S-RNase* mutations result in self-compatible *N. alata*. **(A)** *S-RNase* allele identification in *N. alata*. Primer sets S2F/R and Sc10F/R were used for PCR analysis to determine the presence of *S2* and *Sc10* alleles in the population used for gene editing. **(B)** Cas9-PF was armed with a polycistronic tRNA-gRNA (PTG) cassette consisting of S2 and Sc10 gRNAs. **(C)** Mutation patterns in T0 transgenic plants. sgRNA, single guide RNA; PAM, the protospacer adjacent motif; WT, wild type; inserts and deletions are indicated as ‘i’ and ‘d’ respectively. **(D)** compared to the sterility of WT, well-stacked fruits and mounts of seeds were harvested in two bi-allele edited *N. alata* plants. Scale bar=1 cm. **(E)** Segregation ratio of Cas9-PF-free plants and *S2* and *Sc10* alleles in T1 generation of two bi-allele edited *N. alata* lines respectively.

The SI in *N. alata* is controlled by the highly polymorphic *S*-locus. To identify the *S* allele in our owned line, the degenerate primer pair C2f/C4r (**Supplementary Table 1**) was used to amplify the conserved domains of S-RNases (Roldan et al., 2010). Fragments amplified from random selected 7 plants of the population of *N. alata* were cloned and sequenced. Among 70 sequences (10 clones per plant), 29 and 41 were 100% identical to *Sc10-* and *S2-RNase* respectively, which were previously characterized as functional *SI* alleles (Anderson et al., 1986; Murfett et al., 1996). To further confirm the allelic variants of *S-RNases*, specific primers for *Sc10*- and *S2-RNase* were used to examine the same population. As **Figure 3A** shown, a single band with the expected size was amplified in each case, suggesting the presence of *Sc10-* and *S2-RNases* alleles in plants.

To test whether the developed CRISPR/Cas9 system could be used to target two *S*-*RNases* alleles simultaneously, we designed and armed pairs of gRNA targeting the *Sc10-* and *S2-RNase* first exon to an endogenous tRNA processing system (**Figure 3B**). This system could dramatically enhance the processing efficiency and function of dual sgRNAs transcribed from a single transcript (Xie et al., 2015). According to our optimized transformation method, we generated 26 regeneration plants from 30 explants. To evaluate the efficiency of the target gene editing, we amplified and sequenced the first exons sequences of *Sc10*- and *S2-RNases* gene in all T0 plants. In total, we obtained 12 plants containing mutations in *Sc10- and S2-RNases*. Among them, two plants contain bi-allelic mutations of both *S-RNases*, one contains mutations in *S2-RNase* and four contain mutations in *Sc10-RNase* allele (**Figure 3C**). However, the remaining five mutations were heterozygous in *Sc10-* or *S2-RNases*, which showed overlapping peaks starting at 17th nucleotide of gRNA. We speculated these plants are chimeric editing, which is a common phenomenon in somatic cells gene editing.

To investigate the self-compatibility of the *S-RNase* gene editing plants, we transferred two bi-allelic mutants to the greenhouse under a 16-h light/8-h dark cycle. We performed artificial pollinations at the open flower stage. After self-pollination, compare to the small and shriveled fruit of WT plants, two bi-allelic mutant lines (#11 and #23) produced big and well-stacked fruits (**Figure 3D**). In contrast to the complete sterility of WT plants, self-pollination of two mutant lines produces large amounts of seeds per fruit, ranging from 137 to 349 seeds per fruit. These results demonstrate that inducing loss-of-function mutations in *S-RNase* genes resulted in self-compatibility in *N. alata*.

To seek for the Cas9 cassette free offspring and determine whether the mutations could be inherited to the next generation, we sowed the T1 seeds. The germination rate was > 80% for two lines. The segregation ratio of T1 plants of lines 11 and 23 without the Cas9 cassette is 18.7% and 21.8% respectively (**Figure 3E**). The Chi-square test showed that these two lines contained a single copy of the Cas9 cassette. We further detected mutations in the *S-RNase* genes and genotyping of *Sc10*- and *S2*- allele in all Cas9-free T1 generation plants. The sequencing result showed that bi-allele mutations of *Sc10*- or *S2-RNases* in lines 11 and 23 were transmitted to the T1 generation and all T1 plants harbored mutations in *S2-* and/or *Sc10*-allele. The ratios of *S2S2*, *S2Sc10* and *Sc10Sc10* in the two lines are 19-22%, 52-56% and 22-29%, corresponding to a predictable Mendelian ratio of 1:2:1 (**Figure 3E**). In addition, these mutants have similar plant morphology and growth vigor as the WT (**Supplementary Figure 3**), indicating that they can directly be used for breeding or basic research.

## 4 Discussion

Leaf disk transformation is a widely used method for introducing foreign DNA into plant cells, leading to the development of transgenic plants (Curtis et al., 1995). However, this method may not work for all plant species, even within a plant species, different genotypes or varieties may respond differently to leaf disk transformation. The success of leaf disk transformation is sensitive to the specific parameters of the protocol, such as the choice of tissue culture medium, antibiotics, hormones, and environmental conditions. Variations in these parameters can affect the outcome, and it may be difficult to find the optimal conditions for recalcitrant species. The hypocotyl, located below the cotyledons in the embryonic stem region of a plant, often has characteristics that make it more amenable to transformation. Therefore, hypocotyl transformation is considered a valuable alternative to leaf disk transformation for several reasons. It tends to be less recalcitrant than leaves, making it easier for *Agrobacterium* to infect and introduce foreign DNA. Hypocotyl tissue typically exhibits a higher regeneration potential than leaf tissue in many plant species (Wang and Xu, 2008; Mahto et al., 2018; Muto et al., 2021; Xiao et al., 2022). In this study, we failed to induce callus formation by using leaf disks as explants. However, we observed abundant swelling tissue in *Agrobacterium*-infected hypocotyl segments. By screening different strains of *A. tumefaciens*, we found that EHA105 and GV3101 had relatively high infection efficiency, which is consistent with previous reports in other species.

During the hypocotyl-mediated transformation using EHA105, another challenge was to control *Agrobacterium* contamination in the induced callus and regenerated shoots. Induced calli were easily obtained when we used low concentration cefotaxime, but most of these calli did not survive to the rooting stage due to the incomplete inhibition of *Agrobacterium*. On the other hand, high concentration cefotaxime to control *Agrobacterium* infestation after transformation dramatically suppressed callus induction and shoot regeneration. As timentin is a novel antibiotic that has been utilized in other species’ genetic transformation (Cheng et al., 1998; Krügel et al., 2002), we tested whether timentin could replace cefotaxime in our *N. alata* transformation. We found that timentin dramatically inhibited *Agrobacterium* growth and allowed for regeneration within a wide range of concentrations (**Table 1**). After optimization, the efficiency of genetic transformation achieved more than 80% from <1% as described (Ebert and Clarke, 1990). These results indicate that efficient and repeatable transformation systems were established in *N. alata*. More important, the efficiencies of our optimized transformation system should be high enough to test *Agrobacterium*-delivered CRISPR/Cas9 system for *N. alata* genome editing.

Due to the obvious albino phenotypes associated with *pds* mutation, we selected the first exon and designed a single guide RNA (sgRNA) targeting *NalaPDS* to arm our Cas9-PF vector. According to our optimized transformation method, 23 *NalaPDS* editing events with different size indels were obtained from 43 independent transgenic lines. The percentage of the independent T0 transgenic lines that generated mutations in *NalaPDS* was more than 50% (**Figure 2D**). Moreover, 9 transgenic lines of Cas9-PF*-*NalaPDS showed obvious albino leaf phenotypes. Sequencing results demonstrated that 2 plants harbored homozygous mutations and 7 plants harbored biallelic mutations (**Figure 2D**). These results indicated that targeted mutagenesis using *Agrobacterium*-delivered CRISPR/Cas9 system in *N. alata* is feasible.

*N. alata* is a model species to study gametophytic SI, which is a reproductive barrier that prevents self-fertilization and promotes outcrossing and genetic diversity. SI is controlled by a single polymorphic *S*-locus, which encodes two products: the *S-RNase* in the pistil and the *SLF* in the pollen. If the pollen and pistil share the same *S*-haplotype, the pollen tube growth is arrested by the cytotoxic effect of the S-RNase, resulting in an incompatible reaction. SI plays an important role in genetic diversity in flowering plant evolution, but poses some challenges and limitations to fix useful genetic variation. SI makes the plant always have a highly heterozygous genome, which presents a big challenge to perform high quality haploid genome assembly. Moreover, SI makes it difficult to produce pure lines, which interferes with both basic research and breeding. SI prevents the plant from producing homozygotes, which hampers the development of transgenic or genome-edited plants. SI also complicates the genetic analysis and functional characterization of plant genes, as it can affect the segregation and inheritance patterns of genes in self-incompatible populations (Ye et al., 2018; Enciso-Rodriguez et al., 2019; Lee et al., 2023). Therefore, beyond the *NalaPDS* gene, our research ventured into breaking the SI in *N. alata* by targeting the *S-RNase* gene. As it has at least two different *S-RNases* in *S*-alleles, we additionally wanted to test whether we can edit two genes simultaneously in *N. alata* by using our gene editing system. With a PTG system, we armed the Cas9-PF vector with both *S2* and *Sc10* gRNAs. Ultimately, we obtained two lines with bi-allelic editing events at *S2*- and *Sc10-RNase* from 30 explants.

To test whether the bi-allelic *S-RNase* mutations are enough to break the SI and whether the targeted gene mutagenesis passes to the next generations, two lines were artificially pollinated. Well-stacked fruits and abundant seeds were harvested from both two *s2/sc10* mutation lines, indicating that the mutations were self-compatible. Further analysis of the target gene demonstrated that the mutations had been stably inherited to T1 generation in *N. alata*. The successful knockout of *S-RNase* and the subsequent breakdown of SI in *N. alata* is particularly noteworthy. These *S-RNase* edited lines will be a powerful tool to accelerate basic research and breeding. These self-compatible diploid *N. alata* can be used to produce inbred lines, such as recombinant inbred lines (RILs) or near isogenic lines (NILs), which can be further used for gene mapping and breeding. As more than half of the species of genus *Nicotiana* are SI, the strategy presented should be beneficial for researchers and breeders of other SI wild tobacco.

It is worth mentioning that unlike gene editing in *N. tabacum* and other species by using Cas9-PF, purple phenotype did not occur in *N. alata* (**Figures 1**, **2B**; **Supplementary Figure 2**). As Cas9-PF vector contains a visual selection marker mediated by the constitutive overexpression of PAP1 gene, it was supposed to display distinct purple color in leaf, stem and flower (Liu et al., 2019). However, our results indicated that the *PAP1* gene-mediated visual selection marker was ineffective in *N. alata*. Compared with other *N. alata* lines that showed colorful flowers, the line (PI42334) we used had pure white flowers. Therefore, we speculate that this *N. alata* line may have intrinsic genetic mutation in anthocyanidin synthase pathway that renders the *PAP1*-mediated phenotype less effective. Considering that the anthocyanidin synthase pathway has been proved to be important for biotic and abiotic stress tolerance, it is worth exploring which gene controls the anthocyanidin defect phenotype and whether this gene plays a role in biotic and abiotic stress tolerance in *N. alata*.

The aim of this study was to demonstrate the applicability of the CRISPR-Cas9 system by performing gene knockout of the single *NalaPDS* and allelic *S-RNase* genes using an improved *N. alata* transformation protocol for the first time. A single gRNA was found to be successful to achieve high efficiency editing, resulting in *PDS* mutants with albino phenotypes. Dual gRNA was also proved to be efficient to produce bi-allelic editing, resulting in self-compatible *N. alata*. A rapid, easily operated, highly reproducible, and stable transformation and CRISPR-Cas9-based genome editing system for *N. alata* was established. We expect that the established CRISPR-Cas9 system, with self-compatible lines and improved genetic transformation approach, will enable functional genomics and trait improvement in *N. alata*. In conclusion, this study lays the foundation for a new era of genetic research in *N. alata*.

## Data availability statement

The original contributions presented in the study are included in the article/**Supplementary Material**. Further inquiries can be directed to the corresponding author.

## Author contributions

CY: Data curation, Validation, Writing – review & editing. JMZ: Data curation, Validation, Writing – review & editing. YL: Investigation, Writing – review & editing. HY: Investigation, Writing – review & editing. ZT: Methodology, Writing – review & editing. JDZ: Resources, Writing – review & editing. QG: Resources, Writing – review & editing. ZW: Resources, Writing – review & editing. XS: Formal analysis, Writing – review & editing. BX: Formal analysis, Writing – review & editing. CH: Conceptualization, Data curation, Funding acquisition, Methodology, Project administration, Supervision, Writing – original draft, Writing – review & editing.

## Supplementary material

Supplementary Figure 1 Alignment of genomic DNA and CDs sequences of *PDS* gene in *N. tabacum*, *N. benthamiana*, and *N. attenuata* using the MEGA software. The position of the translation initiation codon is indicated by a black arrow. Conserved primers used for amplifying the first exon of *NalaPDS* are shown by blue arrows. gRNA and the first intron of *NalaPDS* are indicated by red and black underlines, respectively.

Supplementary Figure 2 Genetic transformation of the same Cas9-PF-SRNase plasmid in *N. tabacum* by leaf disk and *N. alata* by hypocotyl segments. In contrast to *N. tabacum* overexpressing *PAP1*, which results in purple-colored calli, leaf and shoot tissue due to the accumulation of anthocyanin, *Agrobacterium*-mediated Cas9-PF-SRNase in *N. alata* does not exhibit any purple coloration. Scale bars = 5 mm.

Supplementary Figure 3 Comparison of morphology and growth vigor between wild type and *S-RNase* gene-edited *Nicotiana alata* plants. WT shows a representative wild type *N. alata* plant, highlighting its standard morphology and growth characteristics under our experimental conditions. *S2Sc10* Gene-edited *N. alata* (*s2sc10*) displays a representative *S-RNase* gene-edited *N. alata* plant, demonstrating the plant’s morphology and growth vigor post-editing. The randomly selected T1 plants of line 11 harbored mutations both in *S2*- and *Sc10*-allele edited plant exhibits characteristics similar to the WT, indicating that the editing did not adversely affect its overall growth and development. Bar = 10 cm.

Supplementary Table 1 Oligonucleotides using in this study.
