# Supplementary figures and images for: Establishment and application of Agrobacterium-delivered CRISPR/Cas9 system for wild tobacco (Nicotiana alata) genome editing
Source: Front Plant Sci. 2024 Mar 4;15:1329697. doi: 10.3389/fpls.2024.1329697 (PMC10944875; doi:10.3389/fpls.2024.1329697)

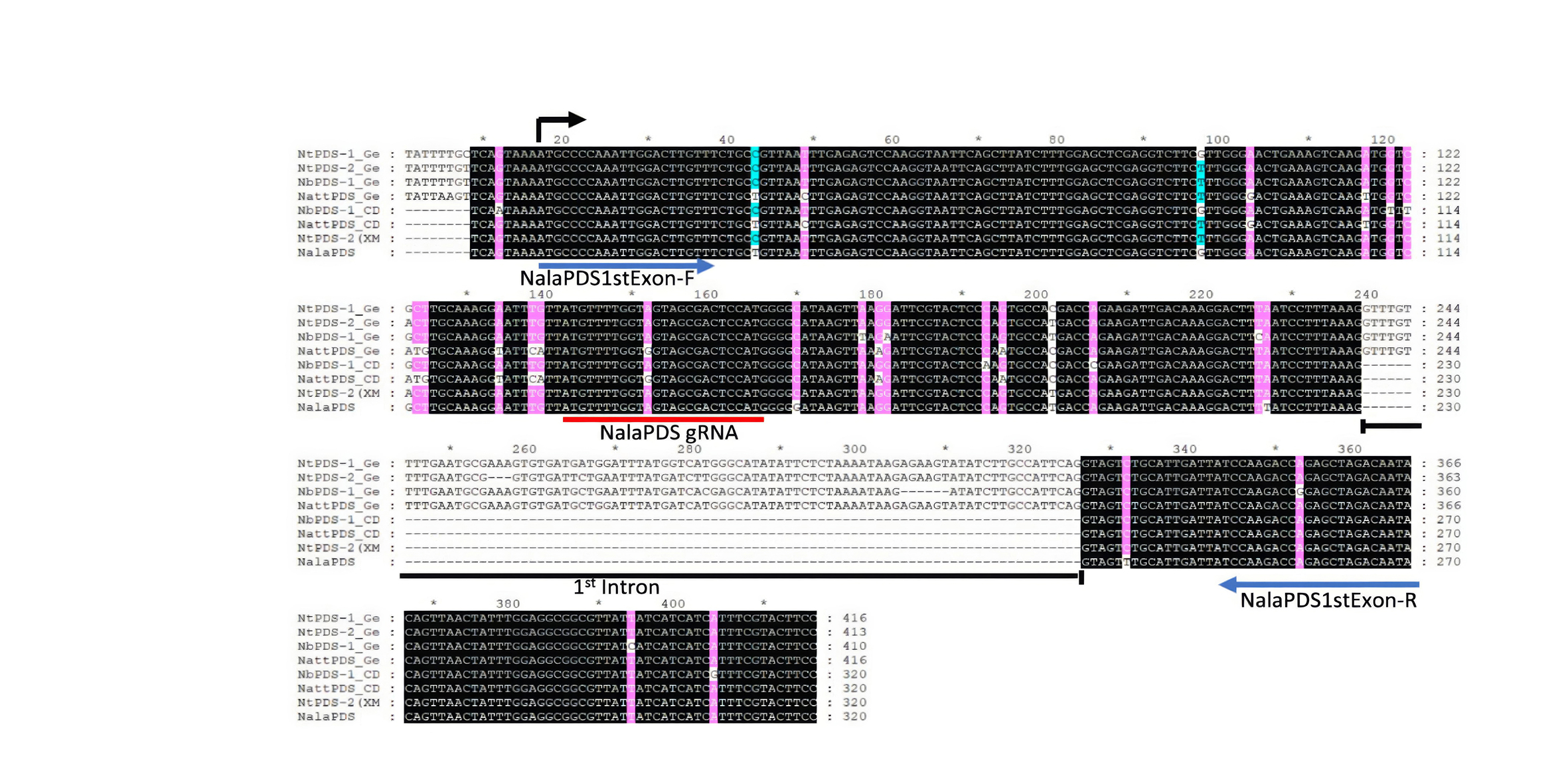

Supplement: Supplementary Figure 1 — Alignment of genomic DNA and CDs sequences of PDS gene in N. tabacum, N. benthamiana, and N. attenuata using the MEGA software. The position of the translation initiation codon is indicated by a black arrow. Conserved primers used for amplifying the first exon of NalaPDS are shown by blue arrows. gRNA and the first intron of NalaPDS are indicated by red and black underlines, respectively. [file Image_1.jpeg]

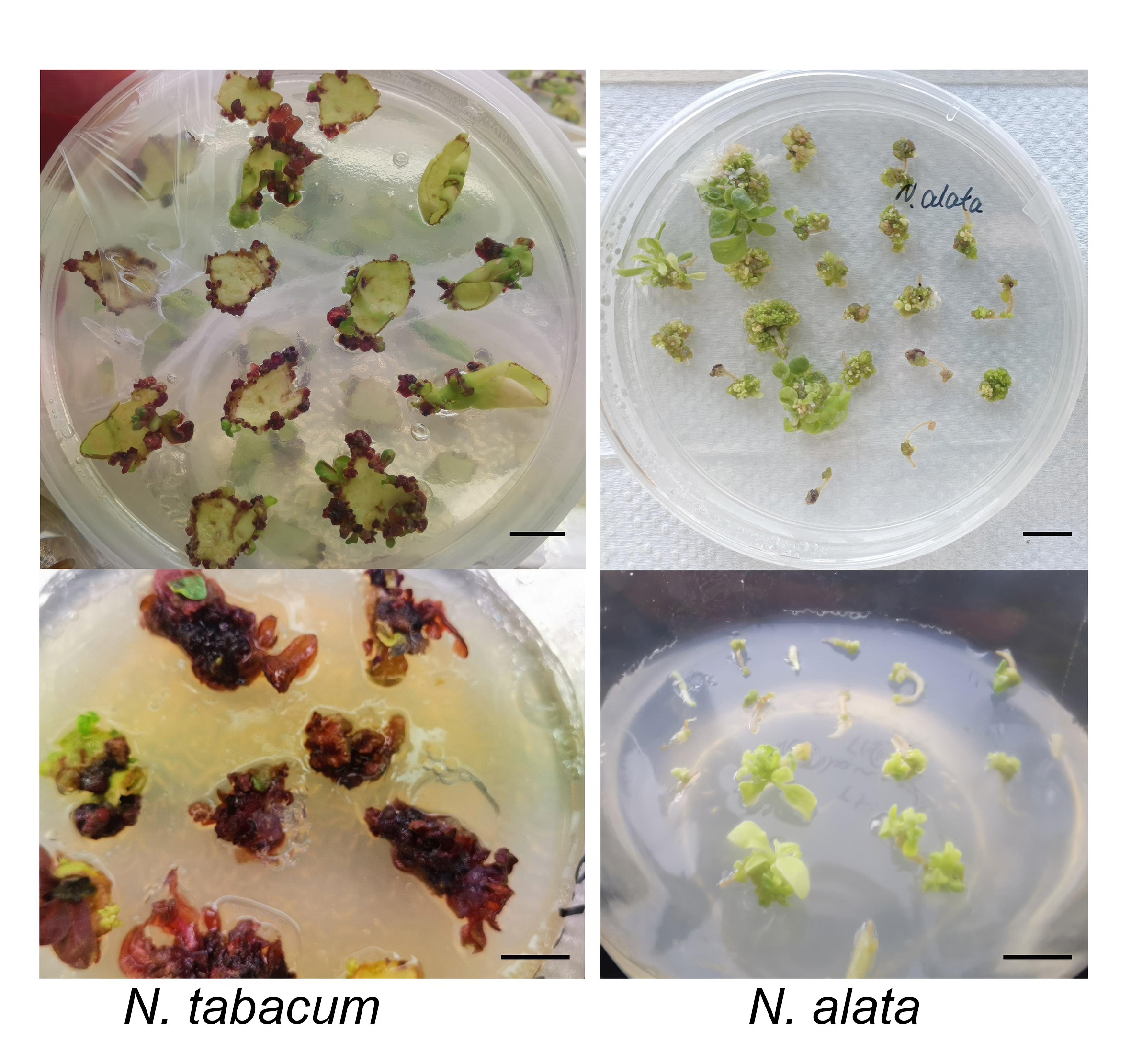

Supplement: Supplementary Figure 2 — Genetic transformation of the same Cas9-PF-SRNase plasmid in N. tabacum by leaf disk and N. alata by hypocotyl segments. In contrast to N. tabacum overexpressing PAP1, which results in purple-colored calli, leaf and shoot tissue due to the accumulation of anthocyanin, Agrobacterium-mediated Cas9-PF-SRNase in N. alata does not exhibit any purple coloration. Scale bars = 5 mm. [file Image_2.jpeg]

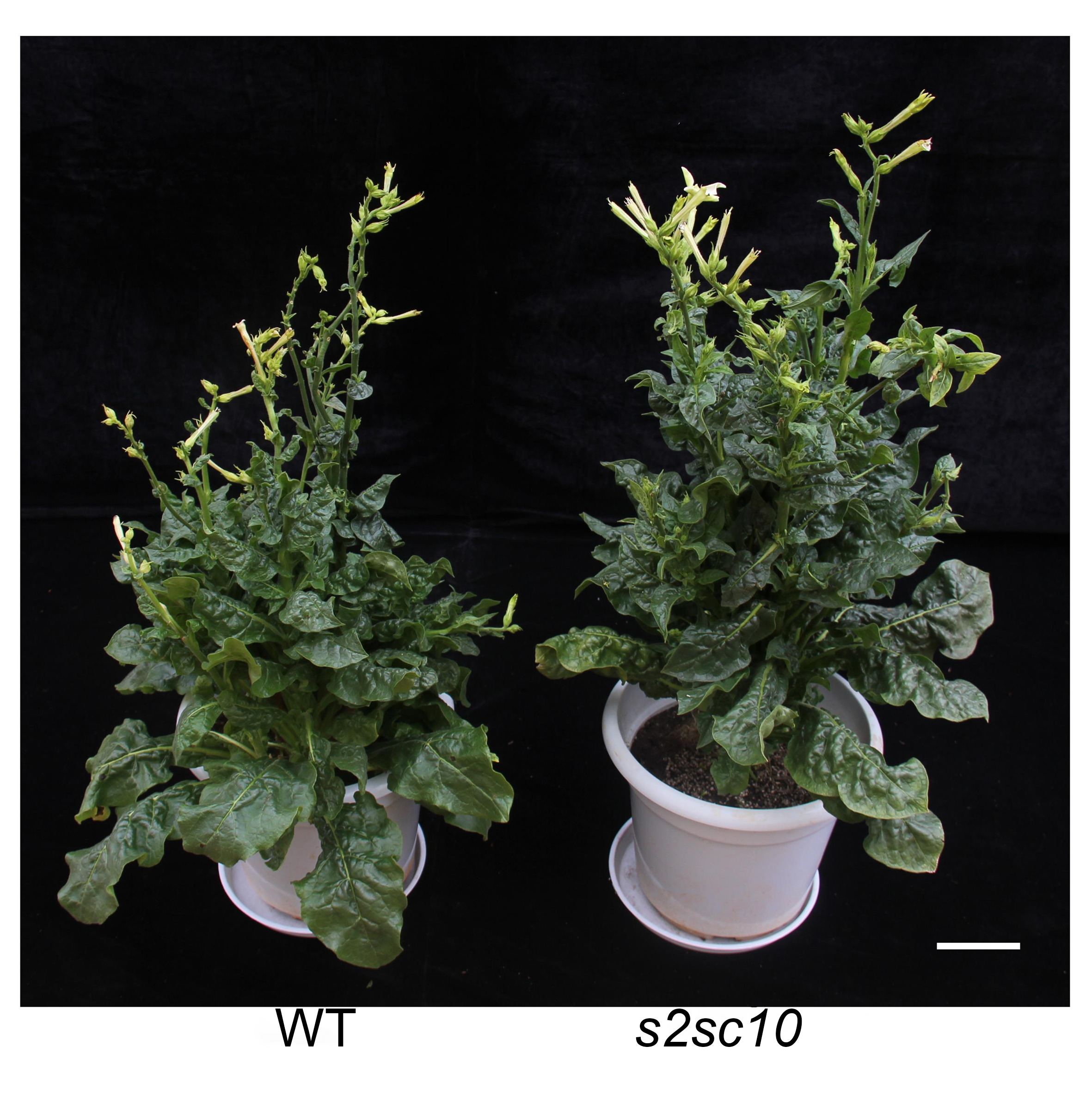

Supplement: Supplementary Figure 3 — Comparison of morphology and growth vigor between wild type and S-RNase gene-edited Nicotiana alata plants. WT shows a representative wild type N. alata plant, highlighting its standard morphology and growth characteristics under our experimental conditions. S2Sc10 Gene-edited N. alata (s2sc10) displays a representative S-RNase gene-edited N. alata plant, demonstrating the plant’s morphology and growth vigor post-editing. The randomly selected T1 plants of line 11 harbored mutations both in S2- and Sc10-allele edited plant exhibits characteristics similar to the WT, indicating that the editing did not adversely affect its overall growth and development. Bar = 10 cm. [file Image_3.jpeg]
