# Supplementary material for: Establishment and application of Agrobacterium-delivered CRISPR/Cas9 system for wild tobacco (Nicotiana alata) genome editing
Source: Front Plant Sci. 2024 Mar 4;15:1329697. doi: 10.3389/fpls.2024.1329697 (PMC10944875; doi:10.3389/fpls.2024.1329697)
Supplement: Supplementary Table 1 — Oligonucleotides using in this study. [file Table_1.docx]

Supplementary Table 1 oligonucleotides using in this study

| oligonucleotide name | Sequence | Purpose |
| --- | --- | --- |
| NalaPDS1stExon-F | ATGCCCCAAATTGGACTTGTT | Amplify the first exon of NalaPDS and test mutations |
| NalaPDS1stExon-R | ACTGTATTGTCTAGCTCTGGTCTTGGA |  |
| NalaPDS sgRNAF | GGCATTGGTAGTAGCGACTCCATG | sgRNA for the NalaPDS ^a^ |
| NalaPDS sgRNAR | CATGGAGTCGCTACTACCAACAAA |  |
| C2f | AACTTTACRATYCAYGRCTTTG | Conserved primers for the S-RNases in *N. alata* |
| C4r | CARAAGATCAAACTTRTCT |  |
| Sc10f | GACCTAACCACCGCAGAAAC | Specific primers for Sc10-RNase |
| Sc10r | CATCGAGTCGAAACATATGCC |  |
| S2f | TTACTGCGATCGCTCCAAACC | Specific primers for S2-RNase |
| S2r | ACAACACGTGCCATGCTTT |  |
| S2SC10PTG | gttgtgcagatgatccgtggcAACAAAGCACCAGTGGTCTAGTGGTAGAATAGTACCCTGCCACGGTACAGACCCGGGTTCGATTCCCGGCTGGTGCATTAGGGTAACAAAATGATGCGTTTTAGAGCTAGAAATAGCAAGTTAAAATAAGGCTAGTCCGTTATCAACTTGAAAAAGTGGCACCGAGTCGGTGCAACAAAGCACCAGTGGTCTAGTGGTAGAATAGTACCCTGCCACGGTACAGACCCGGGTTCGATTCCCGGCTGGTGCAGAACAAAAAGCAATACGAAGgttttagagctagaaatagc | Synthesized polycistronic tRNA-gRNA (PTG) cassette sequence for S2- and Sc10-RNase ^b^ |
| Sc10RNaseTestF | TATCGCGACCAATGGGTAAAAGAC | Editing event test for Sc10-RNase |
| Sc10RNaseTestR | TACAGGTCCTTAGGAAGATAATGTC |  |
| S2RNaseTestF | GGAGAATAAGTTGTCCAAACAGCAG | Editing event test for S2-RNase |
| SA2RNaseTestR | GTGAACATCAGGAAAACAGTTCGTTC |  |

^a^ added 4-nucleotides (letters in red) overhanging at the 5’-end of forward and reverse oligonucleotides used for ligation with *Bsa*I-digested Cas9-PF vector

^b^ Lowercase letters represent sequences that overlapping the linear Cas9-PF vector for homologous recombination, underlined letters represent tRNA sequences, red letters represent gRNA sequences for *S2-* and *Sc10-RNase* and blue letters represent the Spacer sequences.
